# Germline-specific labeling of the somatic chromosomes by protein phosphatase 2A and histone H3S28 phosphorylation in *Acricotopus lucidus*

**DOI:** 10.1007/s00709-017-1092-1

**Published:** 2017-03-06

**Authors:** Wolfgang Staiber

**Affiliations:** Institute of Genetics (240), University of Hohenheim, Garbenstrasse 30, D-70599, Stuttgart, Germany

**Keywords:** Germline–soma differentiation, Orthocladiinae, Germline-specific chromosome marks, Somatic chromosomes

## Abstract

Additional chromosomes limited to the germline (=Ks) were established as a special form of germline–soma differentiation in the Orthocladiinae, a subfamily of the Chironomidae (Bauer and Beermann in Z Naturforsch 7b: 557–563, 1952). The Ks together with the somatic chromosomes (=Ss) pass through a complex chromosome cycle with elimination at mitosis and a monopolar migration of all Ks. The dissimilar behavior of Ks and Ss in these exceptional mitoses initiated the search for differential chromosome marks in the orthocladiid *Acricotopus lucidus*. The search, using immunofluorescence, revealed that in metaphases of male gonial mitoses, and both meiotic divisions, the Ss are fully labeled by protein phosphatase 2A (PP2A) and histone H3S28ph, while in metaphases of somatic cells both marks were detected only at the centromeres of the Ss. In another orthocladiid, *Psectrocladius obvius*, the same labeling pattern of the Ss as in *A. lucidus* was established for H3S28ph, but not for PP2A, which was localised solely at the centromeres. In *Chironomus nuditaris*, a species possessing no Ks, PP2A and H3S28ph signals were always restricted to the centromeres. High levels of H3K4me3, a marker of transcriptionally competent chromatin, were detected on the Ss in metaphases I of *C. nuditari*s, while in both orthocladiids, the Ss in metaphases I were devoid of H3K4me3 signals. This strongly supports an earlier idea of a silencing of the Ss in male meiosis of *A. lucidus* suggesting the possibility of extending this concept to the Orthocladiinae. The germline–soma differentiation in *A. lucidus* is not only made apparent by the occurrence of Ks but also by a germline-specific labeling of the Ss by PP2A and H3S28ph.

## Introduction

Different karyotypes in cells of germline and soma were detected by Bauer and Beermann (1952) in species of the Orthocladiinae, a subfamily of the Chironomidae. A varying number of extra chromosomes in addition to the regular somatic chromosomes (=Ss) was found in germline cells of both sexes. These germline-limited chromosomes (=Ks) are the key players in unusual mitoses occurring in the complex chromosome cycle which they pass through, together with the Ss (Bauer and Beermann 1952; White 1973; Redi et al. 2001). At the stage of the pole cell formation, in the early syncytial embryo, all Ks are eliminated from the future somatic nuclei by remaining in the equatorial plane during mitosis, while the Ss segregate equally. In the first mitosis of the primordial germ cells of newly hatched larvae about half of the Ks are eliminated in the same manner. This reduction in number of Ks is compensated for in the last unequal gonial mitosis by the migration of all Ks unseparated to only one cell pole. The Ss segregate equally in this differential mitosis. The daughter cell getting all the Ks and Ss differentiates into a primary spermatocyte in the male and into the oocyte in the female, while the cell receiving only Ss develops into an aberrant spermatocyte and into a nurse cell. The daughter cells remain connected by a permanent cytoplasmic canal (see diagram in Staiber 2008). The duplication of the Ks by their monopolar migration as unseparated sister chromatids also guarantees that in the primary spermatocyte each of the Ks has a pairing partner in meiotic prophase which again is required for the proper progress of meiosis (Staiber 2014).

Chromosomes restricted to the germline have been detected in a wide variety of animals, such as in the dipteran families of the Cecidomyiidae, Chironomidae and Sciaridae, in hagfish species (Cyclostomata) and in the zebra finch (White 1973; Gerbi 1986; Kubota et al. 1993; Pigozzi and Solari 1998, 2005; Kojima et al. 2010). In *Acricotopus lucidus* (Chironomidae, Orthocladiinae), the Ks have developed from the Ss by endopolyploidisation and rearrangements as established by fluorescence in situ hybridisation using painting probes of the Ss, and have accumulated germline-specific repetitive DNA sequences located in pericentromeric and terminal heterochromatic segments (Staiber and Schiffkowski 2000).

The dissimilar behavior of Ks and Ss in the unusual mitoses in the germline of *A. lucidus* raises the question as to whether there are chromosome markers that are specific for Ks and Ss, or that can differentiate the Ss in germline from those in somatic cells. An immunofluorescence study in *A. lucidus* males revealed an intense labeling of the entire Ss by the protein phosphatase 2A (PP2A) and by the histone H3S28ph in metaphases of gonial mitoses, and in metaphases I and II of meiotic divisions, in contrast to a labeling of the Ss only at their centromeres in metaphases of brain ganglia mitoses. The same labeling pattern of the Ss by H3S28ph, but not by PP2A, was detected in germline and somatic cells of *Psectrocladius obvius*, another species of the subfamily of the Orthocladiinae. Antibodies against PP2A only marked the centromeres of the Ss in metaphases I, and in metaphases of larval brain cells. In *Chironomus nuditaris*, a species of the subfamily Chironominae of the Chironomidae, possessing no Ks, only the centromeric regions of the Ss were labeled by PP2A and H3S28ph in mitotic and meiotic metaphases.

A strong reduction of the level of trimethylated H3K4, a histone modification associated with transcriptionally active or competent chromatin (Schübeler et al. 2004, Muramoto et al. 2010), on the Ss in spermatocyte metaphases I, compared to the high level of H3K4me3 on the Ss in metaphases of the previous gonial mitoses reported earlier for *A. lucidus* (Staiber 2012), was also detected in metaphases I of *P. obvius.* This observation corroborates the idea of a silencing of the Ss in male meiosis of *A. lucidus* (Staiber 2012), and suggests that this situation might also true for all Orthocladiinae.

The present findings demonstrate that in *A. lucidus* the germline–soma differentiation is not only made evident by additional chromosomes limited to the germline but also by the presence of specific PP2A and H3S28ph marks on the somatic chromosomes in the germline.

## Materials and methods

### Animals

Larvae and prepupae of *A. lucidus* (Diptera, Chironomidae, subfamily Orthocladiinae) were taken from a laboratory stock, and those of *P. obvius* (Chironomidae, subfamily Orthocladiinae) and *C. nuditaris* (Chironomidae, subfamily Chironominae) came from wild populations. The latter two species were identified using the polytene chromosome karyotype collection of the Chironomidae of Michailova (1989). In addition, the species identification of *P. obvius* was verified by M. Spies (Bavarian State Collection of Zoology, Munich).

### Chromosome preparation and immunodetection

Chromosome preparations of gonial mitoses, meiotic stages and brain ganglia mitoses were made according to Staiber (2012). Preparations were placed in 0.4% Triton X-100 in phosphate buffered saline (PBS), pH 6.9, for 4 h, blocked with 4% non-fat dry milk in PBS, pH 7.4, for at least 4 h, overlaid with rabbit antibodies for detection of the PP2A-A subunit (polyclonal, Cell Signaling, #2039, Lot 2), PP2A-C subunit (polyclonal, Cell Signaling, #2038, Lot 2), PP2A-B subunit (monoclonal, Cell Signaling, #2290, Lot 1), PP2A-C subunit (monoclonal, Cell Signaling, #2259, Lot 2), H3S28ph (polyclonal, Cell Signaling, #9713, Lot 1, Lot 2), H3S28ph (polyclonal, Millipore, #07-145, Lot 2495654), H3K4me3 (polyclonal, Abcam, #ab8580), H3K4me3 (monoclonal, Cell Signaling, #9751, Lot 8) and with a mouse antibody for detection of the PP2A-C subunit (monoclonal, Millipore, #05-421, lot 2459607) diluted 1:50 to 1:150 in PBS, pH 7.2, with 3% bovine serum albumin (BSA) and 2% normal goat serum (NGS), and incubated in a moist chamber under plastic coverslips at 5 °C for about 40 h. After four washes of 10 min each in PBS-Tween buffer (1× PBS, 0.2% Tween 20, pH 7.4) at room temperature, the primary antibodies were detected using FITC-conjugated goat anti-rabbit IgG or FITC-conjugated goat anti-mouse IgG (Jackson ImmunoResearch, Dianova, Hamburg, Germany) diluted 1:50 in PBS with 3% BSA and 2% NGS at 5 °C overnight. After three final washes in PBS-Tween for 10 min, preparations were embedded in Vectashield mounting medium with DAPI (Vector Laboratories, Burlingame, CA, USA). Images were captured with a Canon 450D digital camera using a Zeiss Axioplan epifluorescence microscope equipped with a Plan-Neofluar 100/1.3 and a Plan-Apochromat 63/1.4 objective and processed with Corel Photo-Paint and CorelDraw software (Ottawa, ON, Canada).

## Results

### Differential labeling of the S chromosomes by PP2A in male germline and somatic cells of *A. lucidus*

The search for differential chromosome marks in the Ss and Ks of *A. lucidus* males using immunofluorescence revealed a germline-specific labeling of the Ss by the phosphatase PP2A. The PP2A holoenzyme is a heterotrimeric complex consisting of a scaffold subunit A (PP2A-A), a regulatory subunit B (PP2A-B), and a catalytic subunit C (PP2A-C). In order to detect PP2A in *A. lucidus*, monoclonal and polyclonal antibodies from different suppliers, produced by immunising animals with synthetic peptides corresponding to the sequence of human PP2A-A, -B or -C subunit proteins, and reported to show cross-reactivity with *Drosophila* or yeast (Millipore, #05-421), were used.

**Fig. 1:**
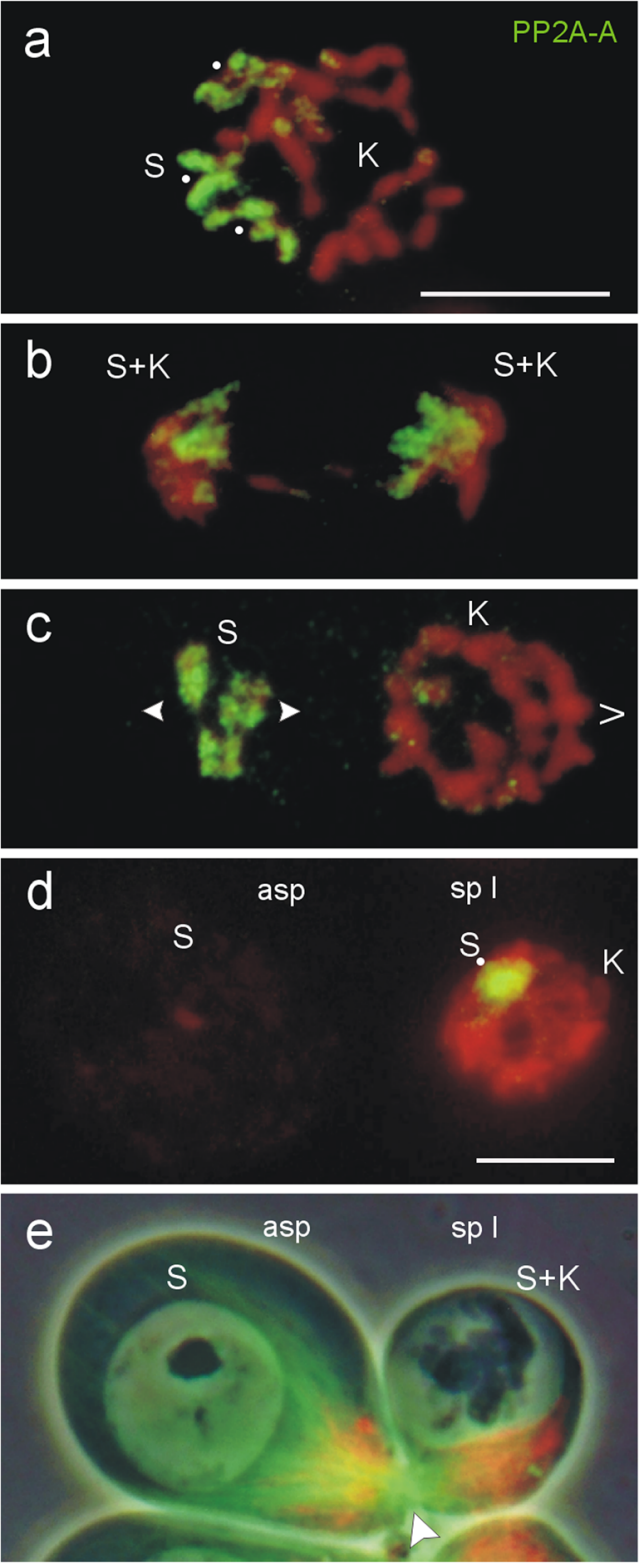
PP2A completely covers the six Ss in germline cells of *A. lucidus* visualised by immunofluorescence using antibodies against the subunit PP2A-A. **a** In a spermatogonial metaphase and **b** an anaphase. **c** In a differential spermatogonial mitosis with monopolar (>) moving Ks and remaining Ss. **d** In an early prophase I nucleus of a primary spermatocyte (*sp I*). The decondensed Ss in the interphase nucleus of the aberrant spermatocyte (*asp*) are devoid of PP2A signals. **e** Primary and aberrant spermatocytes are connected by a permanent cytoplasmic canal (*arrowhead*). Mitochondria (*red*) are transported via the canal from the aberrant to the primary spermatocyte. Microtubules (*green*). Combined fluorescence-phase contrast image. In **a** the paired Ss are marked with *dots*. Chromosomes are stained with DAPI and pseudocoloured red. **a**, **b**, **c**, **e** Same magnification. *Scale bars* = 10 μm

**Fig. 2:**
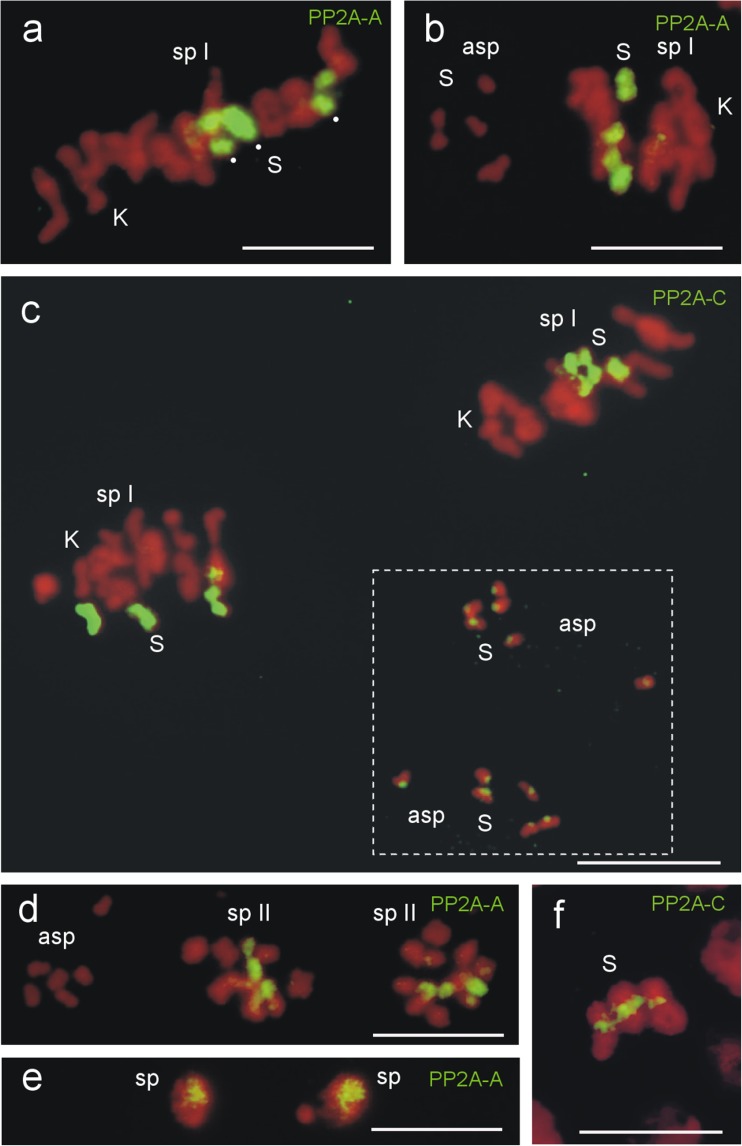
**a**–**d** PP2A labeling of the Ss in meiosis of *A. lucidus* detected by antibodies against the subunits PP2A-A and PP2A-C. **a**–**c** Complete labeling of the three S-bivalents (*dots* in **a**) in metaphases I of primary spermatocytes (*sp I*). **b** No signals were found at the condensed Ss of the aberrant spermatocyte (*asp*) using the polyclonal PP2A-A antibody. **c** The monoclonal PP2A-C antibody detected PP2A at the centromeres of the Ss in both aberrant spermatocytes. The centromeric PP2A fluorescence signals of the Ss in the aberrant spermatocytes were selectively enhanced. The enhanced area is marked by *dots*. For details, see text. **d** Complete PP2A labeling of the Ss in metaphases II of secondary spermatocytes (*sp II*). **e** In the nuclei of young spermatids (*sp*) defined areas exhibit a clear PP2A labeling. Two of the four spermatids are shown. **f** In mitotic metaphases of brain ganglia cells the PP2A signals are limited to the centromeres of the Ss. The homologous Ss are tightly paired. *Scale bars* = 10 μm

Immunostaining with a polyclonal antibody against PP2A-A showed a strong PP2A labeling of the entire Ss in metaphases and anaphases of normal gonial mitosis (Fig. 1a, b), and in differential gonial mitoses of males of *A. lucidus* (Fig. 1c). In meiotic prophase at zygotene/pachytene of the primary spermatocyte, one round area within the nucleus was clearly labeled, while the large interphase nucleus of the affiliated aberrant spermatocyte was devoid of any PP2A signals (Fig. 1d). Both cells are connected by a permanent cytoplasmic canal (Fig. 1e). In metaphases I of the primary spermatocyte, the three S bivalents were completely covered by PP2A-A, while the Ks showed no specific labeling (Fig. 2a, b). The polyclonal PP2A-A antibody gave no signals on the six metaphase-like condensed Ss of the connected aberrant spermatocyte (Fig. 2b), whereas the mouse monoclonal PP2A-C antibody clearly identified PP2A at the centromeres of the Ss in the aberrant spermatocytes (Fig. 2c). However, the PP2A immunofluorescence intensities of Ss bivalents in the metaphases I, and at the centromeres of the Ss in the aberrant spermatocytes, are too different to display this in one image without adjusting the signals. Therefore, in Fig. 2c, the centromeric PP2A fluorescence of the Ss in both aberrant spermatocytes was selectively enhanced (area within the dashed rectangle) to the fluorescence of the S bivalents.

In metaphases II of secondary spermatocytes, the Ss are completely covered by the PP2A-A antibodies (Fig. 2d), and defined areas within the nuclei of young spermatids are also clearly labeled (Fig. 2e). In contrast to the strong PP2A labeling of the whole Ss in germline cells, the PP2A labeling of the Ss in somatic cells, e.g. of the tightly paired homologous Ss in metaphases of brain cell mitoses, by the monoclonal PP2A-C antibody, is limited to the centromeres (Fig. 2f). All antibodies selected for the detection of PP2A, except the monoclonal antibody against PP2A-B, fully labeled the Ss in germline cells of *A. lucidus* males.

### Distribution of PP2A, H3S28ph and H3K4me3 in the S chromosomes in male germline and somatic cells of *A. lucidus*, *P. obvius*, and *C. nuditaris*

**Fig. 3:**
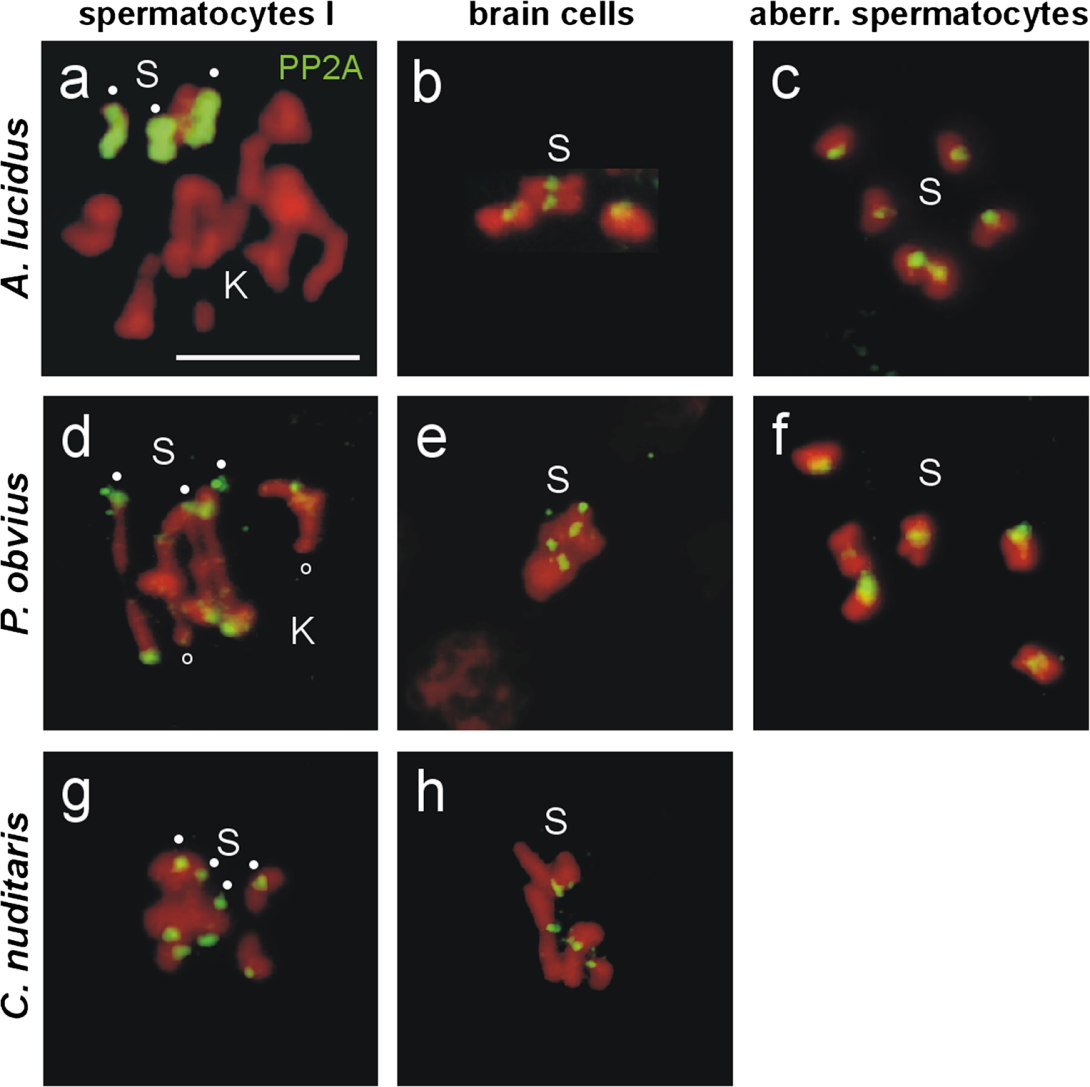
Immunodetection of PP2A in **a**, **d**, **g** metaphases I of primary spermatocytes (S-bivalents, *dots*) and **b**, **e**, **h** mitotic metaphases of larval brain cells of *A. lucidus*, *P. obvius* and *C. nuditaris*, and **c**, **f** aberrant spermatocytes of *A. lucidus* and *P. obvius* using an antibody against PP2A-C. **a** Complete labeling of the entire Ss by PP2A in metaphase I of *A. lucidus*. For further details, see text. *Scale bar* = 10 μm

**Fig. 4:**
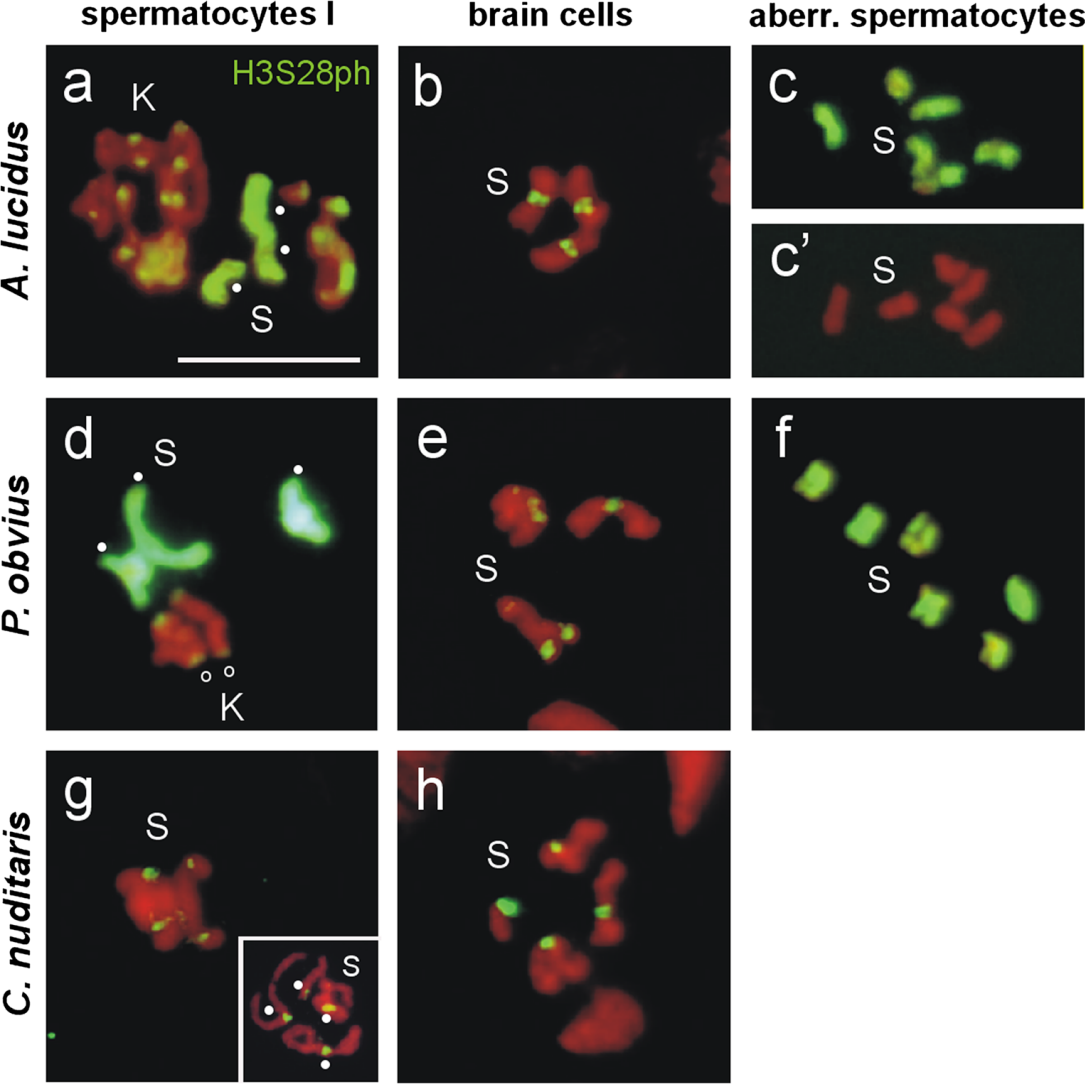
Immunodetection of H3S28ph in **a**, **d**, **g** metaphases I of primary spermatocytes, (S-bivalents, *dots*) and **b**, **e**, **h** mitotic metaphases of larval brain cells of *A. lucidus*, *P. obvius* and *C. nuditaris*, and **c**, **f** aberrant spermatocytes of *A. lucidus* and *P. obvius*. **a**, **d** The Ss of *A. lucidus* and *P. obvius* are completely labeled by the H3S28ph antibody. **g** *Insert*. Prophase I spread of *C. nuditaris* clearly showing the four S-bivalents. For further details, see text. *Scale bar* = 10 μm

**Fig. 5:**
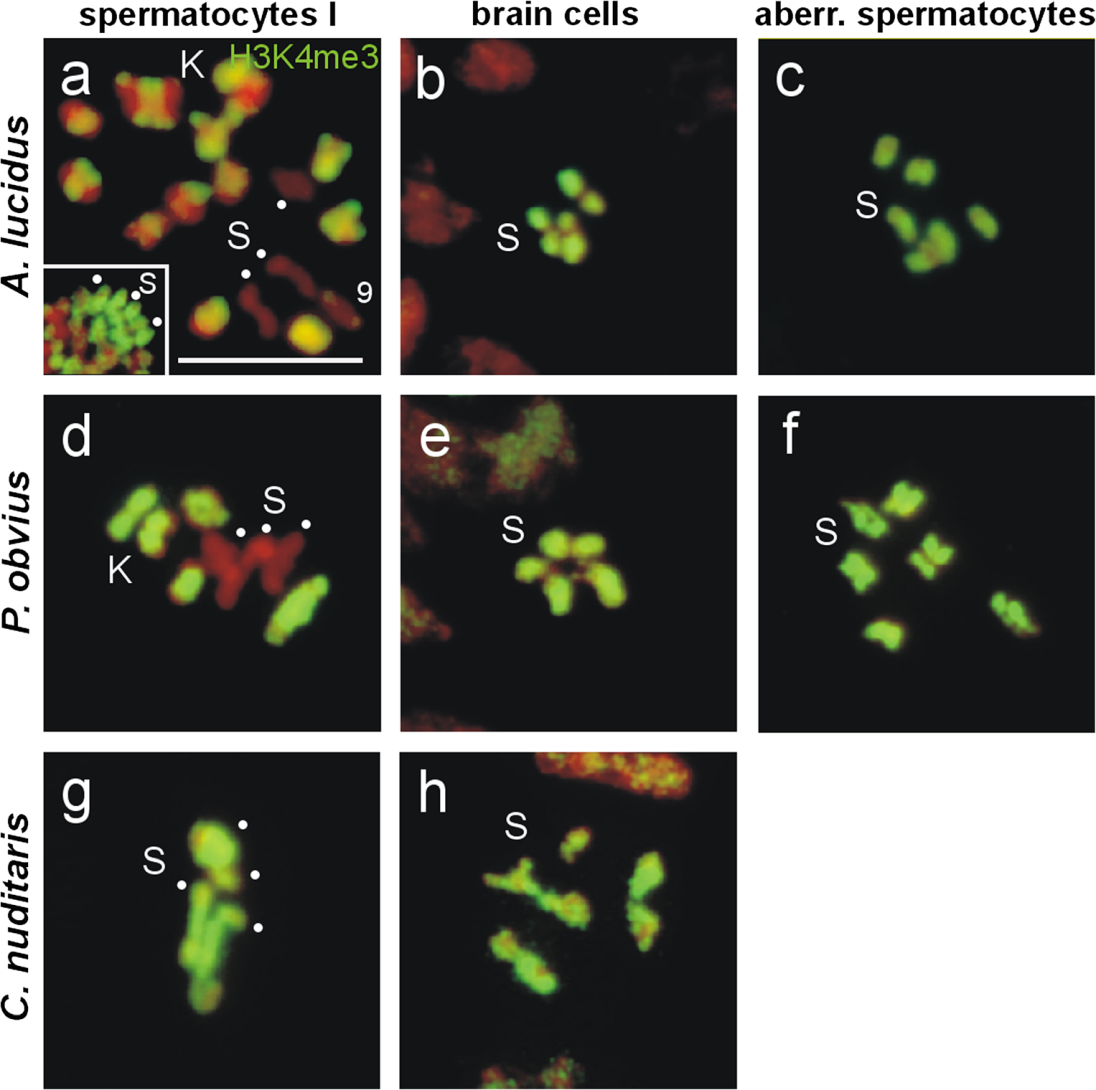
Immunodetection of H3K4me3 in **a**, **d**, **g** metaphases I of primary spermatocytes (S-bivalents, *dots*) and **b**, **e**, **h** mitotic metaphases of larval brain cells of *A. lucidus*, *P. obvius* and *C. nuditaris*, and **c**, **f** aberrant spermatocytes of *A. lucidus* and *P. obvius* **a**, **d** The Ss of *A. lucidus* and *P. obvius* are devoid of H3K4me3 signals. **a** *Insert*. Section of a spermatogonial metaphase with Ss (*dots*) showing intense H3K4me3 labeling. For further details, see text. 9, germline-limited chromosome K9. *Scale bar* = 10 μm

In an earlier study, the labeling of the entire Ss and of the centromeric regions of the Ks in metaphases of male gonial mitoses, and in metaphases I and II of spermatocyte meiosis of *A. lucidus* by H3S28ph antibodies was detected (Staiber 2012). The labeling of the Ss in metaphases of somatic cells was not examined. In the same study, a clear hypomethylation of the Ss regarding H3K4me3 was observed in meiotic metaphases I and II. In order to test whether the differential labeling patterns of the Ss by PP2A, H3S28ph and H3K4me3 are specific for *A. lucidus*, or whether they also occur in other members of the Chironomidae, a further species with Ks, *P. obvius* (subfamily Orthocladiinae), and a species without Ks, *C. nuditaris* (subfamily Chironominae), were investigated (Figs. 3, 4, and 5).

The PP2A labeling of the Ss in spermatocyte metaphases I, in mitotic metaphases of brain ganglia cells and in the aberrant spermatocytes of *A. lucidus*, is shown in Fig. 3a–c. In *P. obvius*, the PP2A labeling of the Ss (2n = 6) in metaphases I, in brain cell metaphases, and in aberrant spermatocytes, was restricted to the centromeres (Fig. 3d–f). The centromeric PP2A signals of the two K-bivalents in Fig. 3d (small circles) are much weaker than those of the S-bivalents. In *C. nuditaris*, the same centromere-restricted PP2A labeling of the Ss (2n = 8) was detected in spermatocyte metaphases I, and in mitotic metaphases of brain ganglion cells (Fig. 3g, h).

Immunodetection with H3S28ph antibodies revealed a strong labeling of the whole Ss in spermatocyte metaphases I, and a labeling of the centromeres of the Ks in *A. lucidus* (Fig. 4a) (Staiber 2012). At diakinesis in the primary spermatocyte, the unpaired Ss in the connected aberrant spermatocyte are still entirely labeled by H3S28ph (Fig. 4c), but they lose this marker rapidly at metaphase I (Fig. 4c’). The paired Ss in mitotic metaphases of brain cells showed H3S28ph antibody signals only at the centromeres (Fig. 4b). In *P. obvius*, the same H3S28ph labeling patterns of the Ss and Ks in metaphases I and of the Ss in ganglion mitoses, as in *A. lucidus*, were detected (Fig. 4d, e). However, the Ss in the aberrant spermatocyte retain their H3S28ph mark (Fig. 4f). In *C. nuditaris*, the Ss in late prophase I (insert in Fig. 4g) and metaphases I (Fig. 4g), and the Ss in metaphases of brain cell mitoses (Fig. 4h) exhibit H3S28ph signals only at the centromeres (Fig. 4g, h).

The histone modification H3K4me3 is associated with transcriptionally active genes and previously active chromatin (Schübeler et al. 2004; Kouskouti and Talianidis 2005; Zhang et al. 2015). In *A. lucidus*, immunofluorescence with H3K4me3 antibodies showed an intense trimethylation at H3K4 of the euchromatic section of the Ks, and a clear hypomethylation of the entire Ss in metaphases I (Fig. 5a). In contrast to metaphases I, in metaphases of the previous spermatogonial mitoses all Ss showed a strong trimethylation of H3K4 (insert in Fig. 5a) (Staiber 2012). The Ss in metaphases of brain ganglia mitoses and in aberrant spermatocytes of *A. lucidus* are fully labeled by H3K4me3 (Fig. 5b, c). In *P. obvius*, the same hypotrimethylation at H3K4 of the Ss and the strong trimethylation of the Ks in metaphases I as in *A. lucidus* was established (Fig. 5d). As in *A. lucidus*, the Ss in brain cell mitoses and in aberrant spermatocytes were completely decorated by the H3K4me3 antibody (Fig. 5e, f). In metaphases I of *C. nuditaris*, however, all Ss are intensely labeled by the H3K4me3 antibody, indicating high levels of trimethylated H3K4 (Fig. 5g). The Ss in brain ganglion metaphases of *C. nuditaris* displayed the same strong H3K4me3 mark (Fig. 5h), as the Ss in ganglion mitoses of *A. lucidus* and *P. obvius*. The results of the labeling of the Ss by PP2A, H3S28ph and H3K4me3 in male germline and somatic cells of *A. lucidus*, *P. obvius* and *C. nuditaris* are summarised in Table 1.

**Table 1:** Summary of the distribution of PP2A, H3S28ph and H3K4me3 in the somatic chromosomes of spermatocytes at metaphase I and brain cells at metaphase of *A. lucidus*, *P. obvius*, and *C. nuditaris* (Chironomidae)

	Germline			Soma		
	Primary spermatocytes			Brain cells		
	PP2A	H3S28ph	H3K4me3	PP2A	H3S28ph	H3K4me3
Orthocladiinae (with Ks)						
*A. lucidus*	+	+	−	●	●	+^a^
*P. obvius*	●	+	−	●	●	+^a^
Chironominae (no Ks)						
*C. nuditaris*	●	●	+^a^	●	●	+^a^

*+* somatic chromosomes fully labeled, *●* only centromeric labeling, *−* no labeling

*Ks* germline-limited chromosomes

^a^Except the centromeres

### Distribution of PP2A and H3S28ph in the S chromosomes in female germline and somatic cells of *A. lucidus*

**Fig. 6:**
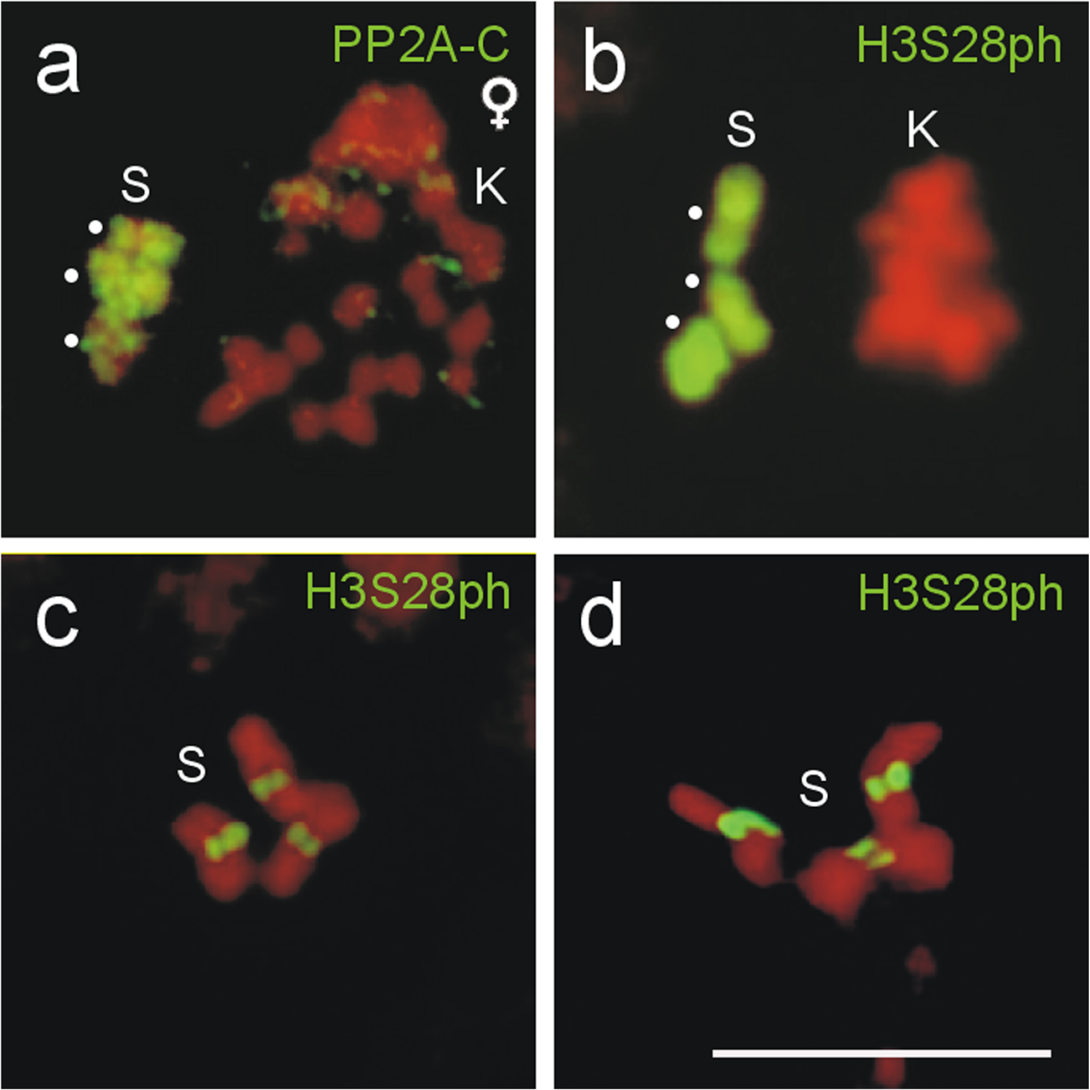
Immunodetection of PP2A and H3S28ph in female germline and somatic cells of *A. lucidus*. In differential oogonial mitoses all Ss (*dots*) are completely labeled by **a** PP2A and **b** H3S28ph antibodies. In mitotic metaphases of **c** brain ganglia and **d** follicle cells PP2A signals are only present at the centromeres of the Ss. *Scale bar* = 10 μm

To answer the question, whether the germline-specific labeling of the Ss occurs in both sexes of *A. lucidus*, metaphases of germline and somatic cells of females were immunostained with PP2A or H3S28ph antibodies. In differential oogonial mitoses, the Ss were fully labeled by PP2A and H3S28ph (Fig. 6a, b), and in metaphases of brain cell and follicle cell mitoses, the H3S28ph labeling was limited to the centromeres of the Ss (Fig. 6c, d). Thus, the specific labeling of the entire Ss by PP2A and H3S28ph in germline cells occurs in males and females of *A. lucidus*.

### Distribution of H3S28ph in the S chromosomes of the aberrant spermatocyte during male meiosis of *P. obvius*

**Fig. 7:**
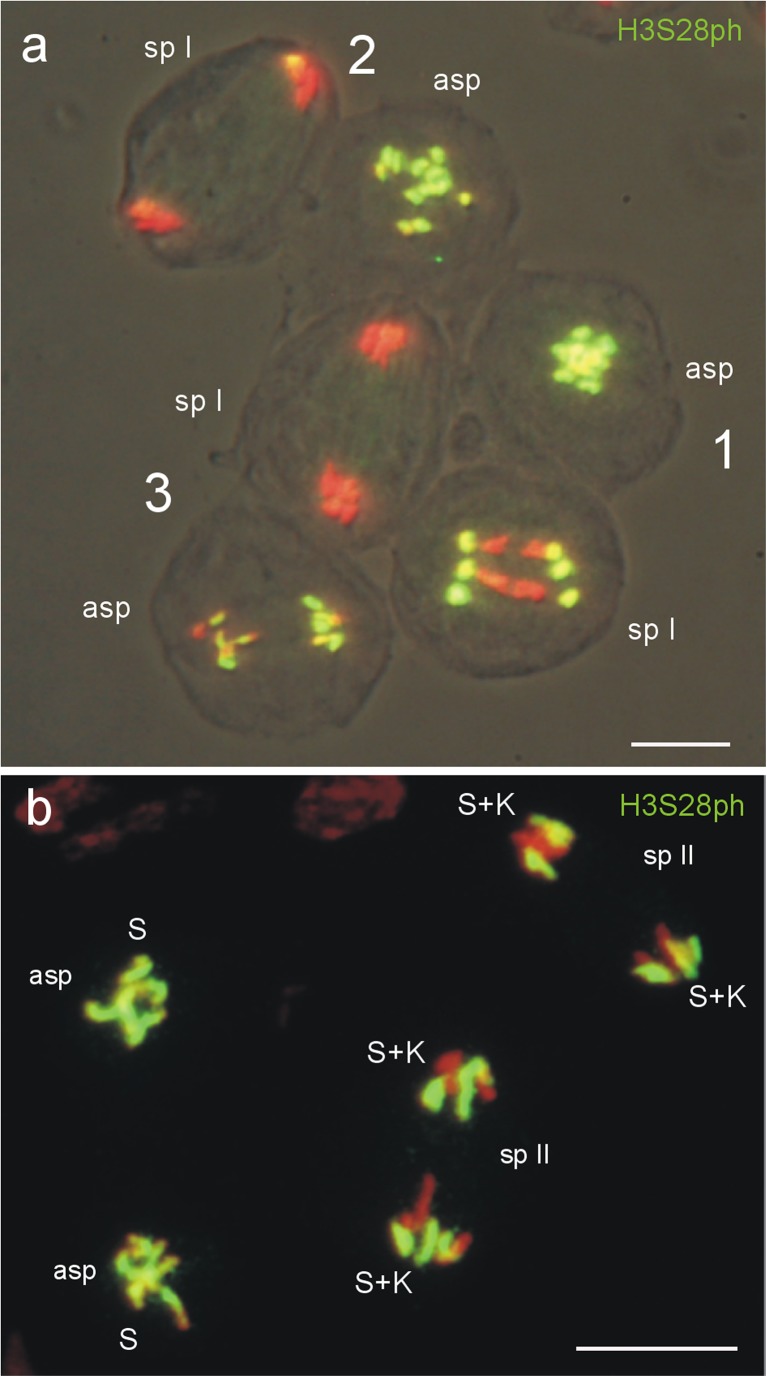
Immunodetection of H3S28ph in regular and aberrant spermatocytes in meiosis of *P. obvius*. **a** Image with three syncytial complexes (*1*–*3*) of primary (*sp I*) and aberrant spermatocytes (*asp*). In mid anaphase I (*1*) of the reduction division in the primary spermatocyte the Ss are still completely labeled by H3S28ph in contrast to the lagging Ks, while in late anaphase I (*2*, *3*) the H3S28ph signals have largely disappeared from the Ss. The connected aberrant spermatocytes passing through mitosis with equal segregation of the six Ss. In early and mid anaphase (*1*, *2*) the Ss are completely labeled by H3S28ph, while in late anaphase (*3*) the mark begins to disappear. **b** In meiosis II the H3S28ph-labeled Ss and the unlabeled Ks of the two secondary spermatocytes (*sp II*) segregate equally, while the two aberrant spermatocytes with the condensed and H3S28ph-labeled one-chromatid Ss remain undivided. *Scale bar* = 10 μm

The development of the aberrant spermatocytes and the labeling of the Ss by H3S28ph differ in the orthocladiids *A. lucidus* and *P. obvius*. In *A. lucidus*, the aberrant spermatocyte remains undivided with Ss arrested in a metaphase-like condensed state during the whole of meiosis of the connected spermatocyte, and the Ss lose the germline-specific H3S28ph labeling at metaphase I (Fig. 4c, c’). In contrast, the aberrant spermatocyte in *P. obvius* undergoes a mitotic division in parallel with the first meiotic division of the primary spermatocyte and the Ss retain the H3S28ph labeling (Fig. 4f). In order to detect whether the Ss of the aberrant spermatocytes in *P. obvius* are also labeled during the second meiotic division of the regular spermatocytes, the distribution of H3S28ph in the Ss was analyzed during the whole of meiosis. Three syncytial complexes (1–3) of dividing primary and aberrant spermatocytes are shown in Fig. 7a. The phosphorylation of the Ss at H3S28 is still clearly visible in mid anaphase I of the primary spermatocyte (sp I of 1, Fig. 7a; the Ss move ahead the Ks), but has almost disappeared in late anaphase I (sp I of 2 and 3, Fig. 7a). The Ss of the dividing aberrant spermatocytes show intense H3S28ph labeling in early and mid anaphase (asp in 1 and 2, Fig. 7a). A reduction of the H3S28 phosphorylation in the Ss becomes visible in late anaphase (asp in 3, Fig. 7a). In meiosis II, the H3S28ph-labeled Ss and the unlabeled Ks of the secondary spermatocytes segregate equally, and migrate poleward, while the presumed one-chromatid Ss in the connected aberrant spermatocyte condense and are intensely labeled by H3S28ph, but do not move (Fig. 7b). In *P. obvius*, in contrast to *A. lucidus*, the condensed Ss of the aberrant spermatocytes are fully labeled by H3S28ph in both meiotic divisions of the connected regular spermatocytes.

## Discussion

In *A. lucidus*, the germline-specific Ks have developed from the Ss by endopolyploidisation and rearrangements (Staiber and Schiffkowski 2000). K-sections translocated by X-rays on or into the Ss of *A. lucidus* showed S-homologous banding patterns in the polytene larval salivary gland chromosomes. The polytenised K-sections closely pair with the homologous section of the Ss and form puffs, and in one case even a Balbiani Ring, indicating that gene sequences of the Ks are conserved and can be activated in a somatic tissue (Staiber and Thudium 1986). The clear homology of Ss and Ks and their dissimilar behavior in the unusual mitoses of *A. lucidus* suggested the presence of specific chromosome markers. PP2A and H3S28ph were identified as such markers.

The phosphatase PP2A plays varied roles in different signaling cascades, and is involved in the regulation of various cellular processes such as cell proliferation, cell division, autophagy and transcription (Janssens and Goris 2001). The PP2A holoenzyme is a heterotrimer composed of the subunits PP2A-A, -B and -C. In mammals, each of the subunits A and C exist in two isoforms α and β. The isoforms of subunit C are highly conserved. With about 20 isoforms, the subunit B is the most diversified subunit of PP2A (Martin et al. 2010). In *Drosophila*, only one PP2A-A and PP2A-C subunit, and three PP2A-B and -B′ subunits have been found (Fischer et al. 2016).

Only in *A. lucidus* the Ss are completely covered by PP2A in germline cells, but not in the other chironomid species investigated. There, PP2A was detected only at the centromeres of the Ss. So far, no case of a labeling of entire chromosomes by PP2A, as in *A. lucidus*, has been reported. This suggests that the germ-line specific PP2A labeling of the whole Ss is a lineage-specific feature of *A. lucidus*. PP2A usually acts via its catalytic activity, though Takemoto et al. (2009) demonstrated that in vertebrates it interacts with condensin II, and functions as a recruiter protein targeting condensin II to chromosomes independent of its phosphatase activity. The function of the high amounts of PP2A covering the entire Ss in the male and female germline of *A. lucidus* is unknown. Thus, it remains to be shown whether PP2A is accumulated as recruiter of other proteins as mentioned before, or due to its phosphatase activity, and whether PP2A is present at the germline Ss in a catalytic active or inactive state.

The centromere-restricted PP2A antibody labeling on the metaphase Ss in brain ganglia mitoses of all species analyzed, and in the Ss in the aberrant spermatocytes of *A. lucidus* and *P. obvius*, results most probably from the detection of PP2A as a component of the cohesin-protecting shugoshin/MEI-S332 - PP2A complex (Kitajima et al. 2006; Nogueira et al. 2014). Quite likely the same applies to the PP2A signals at the opposing directed centromeres of the S and K bivalents in metaphases I of *P. obvius*, and of the S bivalents in metaphases I of *C. nuditaris* (Riedel et al. 2006; Rattani et al. 2013). The low amounts of centromeric PP2A were detected only by a mouse monoclonal antibody against human PP2A-C, which cross-reacts with yeast. It is probable that this antibody recognises a highly conserved epitope on PP2A-C. In *A. lucidus*, no definite PP2A signals could be detected at the centromeres of the Ks in metaphases I, in metaphases of gonial mitoses, or in differential gonial mitosis where they move as unseparated sister chromatids to only one pole. The clear discrepancy in the amount of PP2A present at the centromeres of Ss and Ks suggests differences in the centromeric sister chromatid cohesion between both types of chromosomes.

The phosphorylation of H3S28 by Aurora B kinase increases in early mitosis and is involved in chromosome condensation in mammalian cells (Goto et al. 1999, 2002). Metaphase chromosomes are completely decorated by H3S28ph antibodies (Goto et al. 1999). The dephosphorylation of H3S28ph is done by protein phosphatase PP1 (Goto et al. 2002). In the somatic cells of *A. lucidus* and *P. obvius*, only the centromeres of the metaphase Ss were labeled by the H3S28ph antibody, while the germline Ss in spermatocyte metaphases I and II, and also the Ss in the connected aberrant spermatocytes, were entirely immunostained. Most likely the latter comes about because the aberrant spermatocytes are descendants of the germline.

The histone modification H3K4me3 is typically associated with transcriptionally active genes (Schübeler et al. 2004; Zhang et al. 2015). In a genome-wide microarray analysis of the distribution of histone modifications in more than 40% of the genes of *Drosophila* Kc cells, the transcriptionally active genes were found hypermethylated and the inactive ones hypomethylated for H3K4me2 and H3K4me3 (Schübeler et al. 2004). Furthermore, the level of H3K4 methylation correlated with the level of transcription. H3K4me3 is not only a marker of actively transcribed genes, but can also function as a molecular memory and label of recently active chromatin (Ng et al. 2003; Kouskouti and Talianidis 2005; Muramoto et al. 2010).

High levels of H3K4me3 were detected on the entire metaphase Ss, except for the centromeres, in spermatogonial mitoses of *A. lucidus*. In contrast, the Ss in spermatocyte metaphases I of *A. lucidus* and of the other orthocladiid *P. obvius* were clearly hypomethylated, exhibiting very low levels of H3K4me3. In metaphases I of *C. nuditaris*, a representative of the Chironominae possessing no Ks, the Ss showed high levels of H3K4me3. The Ss in metaphases I of *A. lucidus*, unlike the S-homologous sections of the Ks were also found hypoacetylated at H3K18 and H4K8 (Staiber 2012). Hypoacetylation at this sites, H3K4 hypomethylation and high levels of H3K9me3, are characteristic features of heterochromatin (Richards and Elgin 2002; Li et al. 2008). However, the Ss of *A. lucidus* are not heterochromatic, and show H3K9me3 signals only at their centromeric regions (Staiber 2012). The single germline-restricted chromosome (GRC) of the zebra finch *Taeniopygia guttata* is also clearly hypomethylated at H3K4 in male meiotic prophase, but it is heterochromatic and exhibits high levels of H3K9me3 and of the heterochromatin protein HP1ß (Goday and Pigozzi 2010; Schoenmakers et al. 2010).

The strong trimethylation at H3K4 of the Ss in metaphases of spermatogonial mitoses, compared with the clear H3K4 hypomethylation of the Ss in spermatocyte metaphases I of *A. lucidus* and *P. obvius*, supports an earlier idea of a silencing of the Ss during the period between the last gonial mitosis and the beginning of meiosis in *A. lucidus*, and suggests that this concept could be extended to the Orthocladiinae. Further investigations are necessary to elucidate the purpose and the mechanism of the silencing of the Ss in male meiosis of the Orthocladiinae, and the function of the specific labeling of the entire Ss by PP2A and H3S28ph in the germline of *A. lucidus*.

## References

[CR1] Bauer H, Beermann W (1952). Der Chromosomencyclus der Orthocladiinen (Nematocera, Diptera). Z Naturforsch.

[CR2] Fischer P, Preiss A, Nagel AC (2016). A triangular connection between Cyclin G, PP2A and Akt1 in the regulation of growth and metabolism in *Drosophila*. Fly.

[CR3] Gerbi SA (1986) Unusual movements in sciarid flies. In: Hennig W (ed) Germ-line-soma differentiation. Results and problems of cell differentiation. Springer, New York, pp 71–104 10.1007/978-3-540-39838-7_2 3529273

[CR4] Goday C, Pigozzi MI (2010). Heterochromatin and histone modifications in the germ-line-restricted chromosome of the zebra finch undergoing elimination during spermatogenesis. Chromosoma.

[CR5] Goto H, Tomono Y, Ajiro K, Kosako H, Fujita M, Sakurai M, Okawa K, Iwamatsu A, Okigaki T, Takahashi T, Inagaki M (1999). Identification of a novel phosphorylation site on histone H3 coupled with mitotic chromosome condensation. J Biol Chem.

[CR6] Goto H, Yasui Y, Nigg EA, Inagaki M (2002). Aurora-B phosphorylates histone H3 at serine28 with regard to the mitotic chromosome condensation. Genes Cells.

[CR7] Janssens V, Goris J (2001). Protien phosphatase 2A: a highly regulated family of serine/threonine phosphatases implicated in cell growth and signaling. Biochem J.

[CR8] Kitajima TS, Sakuno T, Ishiguro K, Iemura S, Natsume T, Kawashima SA, Watanabe Y (2006). Shugoshin collaborates with protein phosphatase 2A to protect cohesin. Nature.

[CR9] Kojima NF, Kojima KK, Kobayakawa S, Higashide N, Hamanaka C, Nitta A, Koeda I, Yamaguchi T, Shichiri M, Kohno S, Kubota S (2010). Whole chromosome elimination and chromosome terminus elimination both contribute to somatic differentiation in Taiwanese hagfish *Paramixine sheni*. Chromosom Res.

[CR10] Kouskouti A, Talianidis I (2005). Histone modifications defining active genes persist after transcriptional and mitotic inactivation. EMBO J.

[CR11] Kubota S, Kuro-o M, Mizuno S, Kohno S (1993). Germline-restricted, highly repeated DNA sequences and their chromosomal localization in a Japanese hagfish (*Eptatretus okinoseanus*). Chromosoma.

[CR12] Li F, Huarte M, Zaratiegui M, Vaughn MW, Shi Y, Martiessen R, Cande WZ (2008). Lid2 is required for coordinating H3K4 and H3K9 methylation of heterochromatin and euchromatin. Cell.

[CR13] Martin M, Kettmann R, Dequiedt F (2010). Recent insights into protein phosphatase 2A structure and regulation: the reasons why PP2A is no longer considered as a lazy passive housekeeping enzyme. Biotechnol Agron Soc Environ.

[CR14] Michailova P (1989). The polytene chromosomes and their significance for the systematics of family chironomidae. Acta Zool Fenn, Helsinki.

[CR15] Muramoto T, Müller I, Thomas G, Melvin A, Chubb JR (2010). Methylation of H3K4 is required for inheritance of active transcriptional states. Curr Biol.

[CR16] Ng HH, Robert F, Young RA, Struhl K (2003). Targeted recruitment of Set1 histone methylase by elongating Pol II provides a localized mark and memory of recent transcriptional activity. Mol Cell.

[CR17] Nogueira C, Kashevsky H, Pinto B, Clarke A, Orr-Weaver TL (2014). Regulation of centromere localization of the *Drosophila* Shugoshin MEI-S332 and sister-chromatid cohesion in meiosis. G3 (Bethesda).

[CR18] Pigozzi MI, Solari AJ (1998). Germ cell restriction and regular transmission of an accessory chromosome that mimics a sex body in the zebra finch, *Taeniopygia guttata*. Chromosom Res.

[CR19] Pigozzi MI, Solari AJ (2005) The germ-line-restricted chromosome in the zebra finch: recombination in females and elimination in males. Chromosoma 114:403–409 10.1007/s00412-005-0025-5 16215738

[CR20] Rattani A, Wolna M, Ploquin M, Helmhart W, Morrone S, Mayer B, Godwin J, Xu W, Stemmann O, Pendas A, Nasmyth K (2013). Sgol2 provides a regulatory platform that coordinates essential cell cycle processes during meiosis I in oocytes. elife.

[CR21] Redi CA, Garagna S, Zacharias H, Zuccotti M, Capanna E (2001). The other chromatin. Chromosoma.

[CR22] Richards EJ, Elgin SCR (2002). Epigenetic codes for heterochromatin formation and silencing: rounding up the usual suspects. Cell.

[CR23] Riedel CG, Katis VL, Katou Y, Mori S, Itoh T, Helmhart W, Galova M, Petronczki M, Geran J, Cetin B, Mudrak I, Orgis E, Mechtler K, Pelletier L, Buchholz F, Shirahige K, Nasmyth K (2006). Protein phosphatase 2A protects centromeric sister chromatid cohesion during meiosis I. Nature.

[CR24] Schoenmakers S, Wassenaar E, Laven JSE, Grootegoed JA, Baarends WM (2010). Meiotic silencing and fragmentation of the male germline restricted chromosome in zebra finch. Chromosoma.

[CR25] Schübeler D, MacAlpine DM, Scalzo D, Wirbelauer C, Kooperberg C, Van Leeuwen F, Gottschling DE, O’Neill LP, Turner BM, Delrow J, Bell SP, Groudine M (2004). The histone modification pattern of active genes revealed through genome-wide chromatin analysis of a higher eukaryote. Genes Dev.

[CR26] Staiber W (2008). Centrosome hyperamplification with the formation of multiple asters and programmed chromosome inactivation in aberrant spermatocytes during male meiosis in *Acricotopus*. Cell Tissue Res.

[CR27] Staiber W (2012). Germ-line-limited and somatic chromosomes of *Acricotopus lucidus* differ in distribution and timing of alterations of histone modifications in male gonial mitosis and meiosis. Chromosom Res.

[CR28] Staiber W (2014). GTPase ran strongly accumulates at the kinetochores of somatic chromosomes in the spermatogonial mitoses of *Acricotopus lucidus* (Diptera, Chironomidae). Protoplasma.

[CR29] Staiber W, Schiffkowski C (2000). Structural evolution of the germ-line-limited chromosomes in *Acricotopus*. Chromosoma.

[CR30] Staiber W, Thudium D (1986). X-ray induced rearrangements between germ-line-limited and soma chromosomes of *Acricotopus lucidus* (Diptera, Chironomidae). Genetica.

[CR31] Takemoto A, Maeshima K, Ikehara T, Yamaguchi K, Murayama A, Imamura S, Imamoto N, Yokoyama S, Hirano T, Watanabe Y, Hanaoka F, Yanagisawa J, Kimura K (2009). The chromosomal association of condensin II is regulated by a noncatalytic function of PP2A. Nat Struc Biol.

[CR32] White MJD (1973). Animal cytology and evolution.

[CR33] Zhang T, Cooper S, Brockdorff N (2015). The interplay of histone modifications—writers that read. EMBO Rep.

